# Per- and Poly-fluoroalkyl Substances (PFAS) (Chemicals and Contaminants)

**DOI:** 10.14252/foodsafetyfscj.D-25-00009

**Published:** 2025-03-21

**Authors:** Food Safety Commission of Japan

## Abstract

Food Safety Commission of Japan (FSCJ) conducted a self-tasking risk assessment of per- and poly-fluoroalkyl substances (PFAS) in food. Scientific findings and risk evaluation data regarding PFAS, of international organizations, government agencies in other countries, etc., were reviewed in the current risk assessment. The scientific literature related to three major compounds of PFAS, perfluorooctane sulfonate (PFOS), perfluorooctanoic acid (PFOA) and perfluorohexane sulfonate (PFHxS), was surveyed and served for the discussion. Reference doses were derived from two animal experiments* described below. To determine the reference dose, dose estimation models developed by overseas evaluation institutions were adopted for conversion of POD (point of departure) in animal experiments to POD_HED_ (Human Equivalent Dose). Based on the discussions and estimation, the tolerable daily intake (TDI) was appropriately set as 20 ng/kg body weight/day (2×10^−5^ mg/kg body weight/day) for PFOS and as 20 ng/kg body weight/day (2×10^−5^ mg/kg body weight/day) for PFOA. Insufficient scientific findings precluded the evaluation to specify a reference dose of PFHxS. The average daily intake in Japan was obtained from the Total Diet Study conducted in a limited number of regions during the fiscal years 2012–2014: PFOS (Lower Bound to Upper Bound (LB–UB)** 0.60–1.1 ng/kg body weight/day, and PFOA (LB–UB) 0.066–0.75 ng/kg body weight/day. These values were lower than the TDIs. Due to the lack of sufficient data on PFAS concentrations and their distribution in various foods, it is necessary to be aware of these intake estimates carrying considerable uncertainty.

## Conclusion in Brief

Food Safety Commission of Japan (FSCJ) conducted a self-tasking risk assessment of per- and poly-fluoroalkyl substances (PFAS) in food.

Scientific findings and risk evaluation data regarding PFAS, of international organizations, government agencies in other countries, etc., were reviewed in the current risk assessment. The scientific literature related to three major compounds of PFAS, perfluorooctane sulfonate (PFOS), perfluorooctanoic acid (PFOA) and perfluorohexane sulfonate (PFHxS), was surveyed and served for the discussion.

The items for this discussion were extracted from the evaluation reports of overseas assessment agencies. Then the possible effects were reviewed endpoint by endpoint and determined whether they were adequate for evaluating adverse health effects.

Regarding non-carcinogenic health effects, the following four outcomes reported in epidemiological studies for PFOS and PFOA were evaluated: increase in serum ALT levels, increase in serum total cholesterol levels, decrease in birth weight, and decrease in antibody responses following vaccination. These data were, however, not selected in the present discussion as endpoints for the following reasons.

1. Serum ALT and total serum cholesterol levels were associated with PFOS and PFOA. The serum levels increased slightly, but the clinical significances were not evident due to the lack of their links with the onset of diseases afterwards. No clear relationships existed between levels of the clinical parameters and of PFOS or PFOA as the whole.

2. Antibody response after vaccination was also one of the endpoint candidates but the influence remained undefined.

3. Data of PFOS or PFOA on the effects of birth weight reduction, such as small for gestational age (SGA) and low birth weight (< 2,500 g), were reported, but limited, and thus judged not to reach consensus findings. Furthermore, the impact of PFOS or PFOA on postnatal growth remained unclear.

The carcinogenicity was evaluated as follows and was not adopted as an endpoint:

1. In animal studies; increases in hepatocellular tumors in rats exposed to PFOS, and Leydig cell tumors, hepatocellular adenomas, hepatocellular carcinomas, and pancreatic acinar cell adenomas in rats exposed to PFOA were observed. The detailed mechanisms were unclear for carcinogenicity and possible to relate to the rodent’s specific activation of PPARα. FSCJ thus considered the results in rats might not be directly extrapolated to humans.

2. Associations of PFOA exposure with kidney cancer, testicular cancer, and breast cancer were reported, but not consistent among epidemiological studies. The data regarding the associations between PFOS and breast cancer and between PFHxS and kidney or breast cancer were judged insufficient.

Reference doses were derived from two animal experiments* described below. To determine the reference dose, dose estimation models developed by overseas evaluation institutions were adopted for conversion of POD (point of departure) in animal experiments to PODHED (Human Equivalent Dose).

Based on the following discussions and estimation, the tolerable daily intake (TDI) was appropriately set as 20 ng/kg body weight/day (2×10^−5^ mg/kg body weight/day) for PFOS and as 20 ng/kg body weight/day (2×10^−5^ mg/kg body weight/day) for PFOA. Insufficient scientific findings precluded the evaluation to specify a reference dose of PFHxS.

1. PFOS: The suppressed body weight gain in offspring observed in a two-generation reproductive and developmental toxicity study in rats^1^^)^ was adopted for the endpoint. The reference value of health effects was estimated using the calculation methods of PODHED and RfD by U.S. Environmental Protection Agency (U.S. EPA), Food Standards Australia New Zealand (FSANZ), and Agency for Toxic Substances and Disease Registry (ATSDR). NOAEL converted to NOAELHED of 0.0005–0.0006 mg/kg body weight/day and an uncertainty factor of 30 (Uncertainty factors for interspecies difference of 3 and intraspecies difference of 10 in toxicodynamics. No toxicokinetic factor was added because it was included in the dose estimation model.) provided the reference value of 20 ng/kg body weight/day.

2. PFOA: The reduced number of ossification sites in the proximal phalanges of the forelimbs and hindlimbs of fetuses, the acceleration of sexual maturation in male offspring observed in mouse reproductive and developmental toxicity studies^2^^)^ were adopted for the endpoint as with U.S. EPA. LOAEL of 1 mg/kg body weight/day was also adopted for POD. PODHED of 0.0053 mg/kg body weight/day calculated by U.S. EPA and Uncertainty Factor of 300 (Uncertainty factors for interspecies difference of 3 and intraspecies difference of 10 in toxicodynamics, and for LOAEL use of 10. No toxicokinetic factor was added because it was included in the dose estimation model.) provided the reference value of 20 ng/ kg body weight/day.

With sufficiently increased knowledge on PFAS, including the clinical significance of the reported health effects and dose-response relationships, revising the TDI may be possible in the future.

The average daily intake in Japan was obtained from the Total Diet Study conducted in a limited number of regions during the fiscal years 2012–2014: PFOS (Lower Bound to Upper Bound (LB–UB)** 0.60–1.1 ng/kg body weight/day, and PFOA (LB–UB) 0.066–0.75 ng/kg body weight/day. These values were lower than the TDIs.

Due to the lack of sufficient data on PFAS concentrations and their distribution in various foods, it is necessary to be aware of these intake estimates carrying considerable uncertainty.
